# Polyadenylation of ribosomal RNA by *Candida albicans *also involves the small subunit

**DOI:** 10.1186/1471-2199-5-17

**Published:** 2004-10-04

**Authors:** Jacob Fleischmann, Hong Liu, Chieh-Pin Wu

**Affiliations:** 1 Department of Medicine, Veterans Affairs Greater Los Angeles Healthcare System, UCLA School of Medicine, 11301 Wilshire Boulevard, Los Angeles, California 90073 USA; 2 Department of Oral Biology and Medicine, UCLA School of Dentistry, University of California, 10833 Le Conte Ave. Los Angeles, California 90095, USA

## Abstract

**Background:**

*Candida albicans *is a polymorphic fungus causing serious infections in immunocompromised patients. It is capable of shifting from yeast to germinating forms such as hypha and pseudohypha in response to a variety of signals, including mammalian serum. We have previously shown that some of the large 25S components of ribosomal RNA in *Candida albicans *get polyadenylated, and this process is transiently intensified shortly after serum exposure just prior to the appearance of germination changes.

**Results:**

We now present data that this process also involves the small 18S subunit of ribosomal RNA in this organism. Unlike the large 25S subunit, polyadenylation sites near the 3' end are more variable and no polyadenylation was found at the reported maturation site of 18S. Similar to 25S, one or more polyadenylated mature sized 18S molecules get intensified transiently by serum just prior to the appearance of hypha.

**Conclusions:**

The transient increase in polyadenylation of both the large and the small subunits of ribosomal RNA just prior to the appearance of hypha, raises the possibility of a role in this process.

## Background

*Candida *species are now among the most important pathogens especially for the immunocompromised host. They are the fourth most common organisms recovered from blood cultures in hospitalized patients [1]. *Candida albicans *the most frequently isolated of the species, is a polymorphic organism. It can switch from a yeast form (blastospore) to a filamentous phase (hypha and pseudohypha) in response to a variety of external stimuli, including mammalian serum. Mutants defective in this serum response, also show a reduced capacity to cause disease in a murine model, [2] suggesting a virulence role for it.

It is widely accepted that the production of the RNA components of ribosomes in eukaryotes proceeds through the transcription of large pre-RNA molecules by RNA polymerase I (Pol I), that get processed into the final large and small subsegments [3]. We have recently reported the unexpected finding that *Candida albicans *polyadenylates some of its 25S ribosomal RNA (rRNA) and the polyadenylation site corresponded to the large subsegment 3'-end maturation [4]. We also found that the concentration of the polyadenylated form of 25S was increased transiently by serum just prior to the appearance of filamentous forms, raising the possibility for a role in hyphal transformation. A question raised by these data was whether this event is unique for the large subunit rRNA or it represents a wider function. For example, is a DNA sequence upstream from the 25S subunit functioning serendipitously as a promoter for RNA polymerase II (Pol II), allowing transcription by this enzyme complex and subsequent polyadenylation. Such a process would not likely be important for ribosomal function. Similar involvement of other subunits on the other hand, would increase the likelihood that polyadenylation of rRNA plays a wider role in the biology of this yeast. We now report our observations related to the 18S subunit of rRNA, that indeed there are other polyadenylation sites located near the 3'end of the 18S subunit. Similar to 25S, we found that a polyadenylated 18S transcript, similar in size to a processed mature molecule, is also enhanced early and transiently by serum, further strengthening the possibility of a regulatory role for polyadenylation in the germination process.

## Results

### Cloning of poly A -extended 18S subsegments

**Figure 1:**
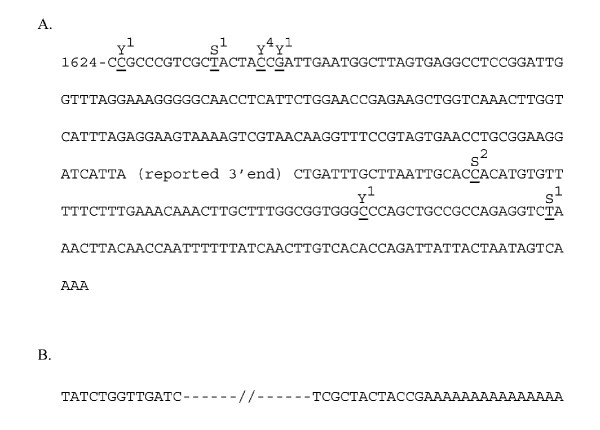
**Polyadenylation sites of 18S rRNA subunit and a full length polyadenylated 18S clone. **Polyadenylation positions on 18S subunit. (A) represents the clones derived from amplification with primers designed for the 3'end of 18S. Underlined letters represent polyadenylation sites. Y or S over them indicate whether RNA came from yeast exposed to YPD (Y) or serum (S) and superscripted digits over them indicate the number of clones found at that position. The bold enlarged letters represent the sequence with the most polyadenylation sites. The #1624 represents the position from 5' end of 18S. (B) represents partial sequences at the 5' and 3' ends of a full length clone of a polyadenylated 18S molecule.

We have found polyadenylation to occur both in yeast grown in YPD and in those exposed to serum for 5 minutes. In all cloned PCR products the number of adenines in the chain exceeded those in the poly-T primer (DT12) used to generate them. Furthermore, the anchor sequence assures us that we did not extend an inappropriately annealed primer. Unlike our data with the 25S subunit, where the attachment was to one of two thymidines one base apart at the reported maturation site [4], the polyadenylation sites near the 3' end of 18S subunit were congregating either upstream or downstream of the reported maturation site [5] (Figure 1A) but none were at the reported site. Six of seven YPD exposed yeast polyadenylation sites were between positions 1625 and 1643, located 148 bases upstream from the reported 3' end, whereas 3 of 4 polyadenylation sites downstream from the maturation site were from yeast exposed to serum. We were able to amplify a full-length clone from YPD exposed yeast and its site of polyadenylation was at position 1643 (Figure 1B) near the other polyadenylation sites of yeast exposed to YPD.

### Serum enhancement of polyadenylation

**Figure 2:**
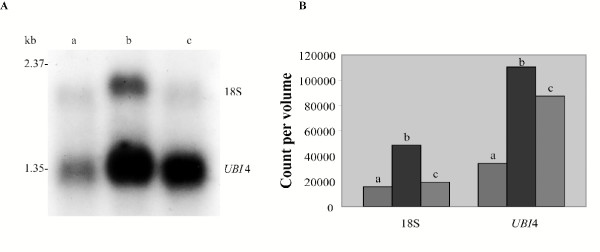
**Upregulation of 18S rRNA polyadenylation by serum exposure. **Northern blot of poly-A selected RNA hybridized by 18S and *UB*14 specific probes (A). (a) is RNA derived from cells grown in YEPD at 30°C, (b) is RNA from yeast in serum for 5 minutes at 37°C, and (c) is RNA from yeast in serum for 15 minutes at the same temperature. (B) represents the quantitative phosphorimager data of the Northern shown in (A). Small letters (a, b, c) are the same as in (A).

Similar to 25S, polyadenylation of 18S subunit was enhanced by serum exposure and this is shown in Figure 2A, representing a Northern blot utilizing poly-A selected RNA, hybridized with an 18S specific probe. By 5 minutes the intensity of the 18S band was more than tripled but back to baseline levels at 15 minutes. *UBI4 *was also up-regulated by serum but its intensity remained the same at 15 minutes, while the 18S band returned to baseline assuring us that the 18S enhancement was not as a result from an error in RNA loading. We previously found *ACT1 *[4] to show the same pattern, suggesting that serum exposure may also up-regulate constitutive genes. As a control for temperature, we also exposed yeast to YPD at 37°C and to serum at 30°C. There was no increase in polyadenylation in YPD at 37°C and there was increase with serum exposure at 30°C (data not shown) indicating that serum caused this increase. Estimates from phoshporimager data indicate that at baseline in YPD less than 1% of the rRNA is polyadenylated (data not shown). Polyadenylation of rRNA in *Saccharomyces cerevisiae *has recently been described in similar amounts [6].

**Figure 3:**
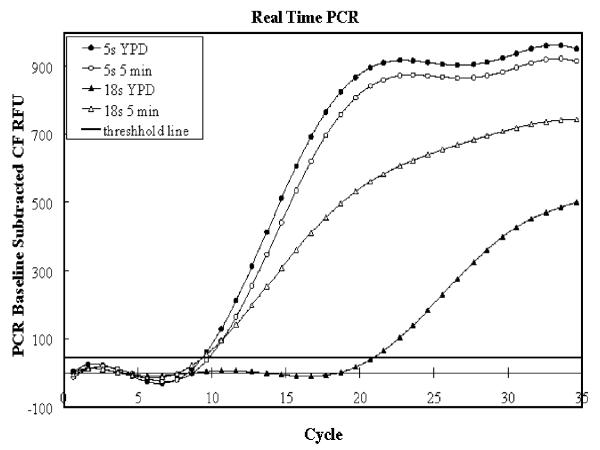
**Real-time PCR confirmation of 18S rRNA polyadenylation upregulation by serum. **Real-time PCR reactions represented as PCR baseline subtracted relative fluorescence units (RFU) versus cycle number plots. Triangles represent reactions with 18S specific primers and circles represent reactions with 5S specific primers.

To further confirm that the 18S increase from time zero to 5 minutes is real, we performed real-time PCR reactions and the results are shown in Figure 3. As can be seen, amplification can be detected 10 cycles earlier when the template is derived from organisms exposed to serum, confirming the increased amount of 18S in the starting material. 5S is detected at the same cycle whether exposed to serum or not.

The 18S band in Figure 2A, lane b is slightly up-shifted as compared to lanes a and c, suggesting that likely one or more molecules, whose polyadenylation sites are downstream from the reported maturation sites are up-regulated by serum. This is consistent with our findings above, that 3 of the 4 polyadenylation sites located in that region were from serum exposed yeast and it suggests that serum up-regulation of polyadenylation may be selective to these downstream sites. When the same filter was hybridized with the 5S specific probe no bands were detected, indicating that our 18S bands were not a result of rRNA contamination (data not shown). Figure 2B represents the phosphorimager generated counts of these bands confirming the visual results objectively. Northerns that included the polyA-minus fractions (data not shown) continue to show the 18S bands indicating that rRNA transcripts with and without polyadenylated extensions are being produced.

## Discussion

These data indicate that 18S subunit mirrors the large 25S molecule as regards to polyadenylation and its response to serum, suggesting that this is not an incidental phenomenon. They do differ from the 25S subunit in that polyadenylation occurs both upstream and downstream to but not at the reported 3'-end maturation site, while for the 25S subunit, the polyadenylation was found to be exclusively at the maturation site. Perhaps the 3'-end of the 18S subunit plays an important role in the recognition of start sites on mRNA, and is vigorously protected from modifications. This would also suggest that polyadenylation of 18S has a role outside of the ribosome.

One of the basic questions raised by our original data was whether these polyadenylated transcripts are products of Pol I and get polyadenylated following maturation cleavage or are newly transcribed by Pol II. These data do not resolve this question. While finding of multiple polyadenylation sites in 18S with most of them clearly not corresponding to a reported maturation site, might result from transcription by an enzyme other than Pol I, it is just as likely that they may represent inappropriate cleavage by the ribosomal RNA processing apparatus and these products are being readied for degradation by polyadenylation. The recent report of polyadenylation in *Saccharomyces cerevisiae *that was found to be increased in mutants lacking the degradative function Rrp6p [6] favors the latter scenario. RNA polymerase switching from Pol I to Pol II for rRNA transcription has been described for *Saccharomyces cerevisiae *[7] in cells where the gene for one of the components of the Pol I transcription factor UAF (upstream activation factor) was mutated. These mutants gave rise to isolates that were utilizing Pol II for their rRNA transcription and this newly switched-on state was heritable even through meiosis. These data indicate that *S. cerevisiae *has the inherent capacity to utilize Pol II for rRNA transcription but that this capacity is suppressed by a mechanism that includes UAF. These mutants though, switched to Pol II transcription exclusively. Our data with *C. albicans *differs in that both polyadenylated and non-polyadenylated forms are produced simultaneously. Conrad-Webb and Butow [8] have described rRNA transcripts of various lengths that were polyadenylated, produced by a respiratory-deficient isolate of *S. cerevisiae*. The template utilized by this strain was an episomal copy of ribosomal DNA that contained a Pol II promoter sequence overlapping with the Pol I promoter. Recently, circular and linear rDNA plasmids have been reported in *C. albicans *[9] for the first time. Thus it is possible that one of these episomal elements also contains Pol II promoters allowing it to function as the template for polyadenylated forms of rRNAs. With our findings that polyadenylation also involves 18S, such Pol II promoters would have to be present for both subunit genes making Pol II role less likely. Polyadenylation of a small percentage of total RNA in *Escherichia coli *has been reported [10,11] including rRNA and this polyadenylation occurred even in wild type organisms. Hence it appears, that polyadenylation of these stable molecules occurs more widely as a biological phenomenon.

The role of polyadenylation of rRNA in *C. albicans *is unknown. Open reading frame analysis of the 18S subunit indicates that translation into protein is unlikely, as it would result in peptides shorter than 40 amino acids. Multiple polyadenylation sites upstream and downstream from the reported maturation site suggest, that these may be inappropriately processed molecules that are being readied for degradation, though one of the downstream sites may be an A2 processing site. The mere up-regulation of polyadenylation by serum prior to germination also does not indicate a role in hypha formation as other genes such as ubiquitin and actin also respond similarly. There are aspects to our new 18S and our previous 25S data that leave the possibility for a role in germination open. These include the transient nature of this up-regulation for both subunits just prior to germination and the possible selective nature of this process involving 18S.

## Conclusions

The ribosome is central to cellular function and the RNA component of this organelle assumes critical structural and catalytic roles. Our initial unexpected finding of polyadenylation of a portion of the large rRNA subunit is now extended to the small subunit. That this modification also involves the other major component of the ribosome points to a biological role for this process. The fact that the transient up-regulation of RNA polyadenylation from both subunits just precedes the phenotypic expression of germination, suggests a possible role in regulating *Candida albicans' *polymorphic behavior.

## Methods

### Organism and germination conditions

*Candida albicans *SC5314 (obtained from W. Fonzi) [12] was grown in YPD medium (1%, w/v, yeast extract; 2%, w/v, peptone; 2%, w/v dextrose) at 30°C. Heat inactivated (56°C for 30 minutes) fetal bovine serum (FBS) (10%, v/v in H_2_O) was utilized to induce germination. Yeast cells were grown overnight in YPD at 30°C, harvested by centrifugation, washed once in H_2_O and transferred to FBS pre-heated to 37°C at 1–5 × 10^6 ^cells ml^-1^

### RNA isolation

RNA from cells at various growth conditions was obtained as follows. Incubating mixtures were rapidly cooled in an ice-water bath and were thereafter centrifuged at 4°C and washed with ice cold water once. Cell walls were digested by suspending the pellet in a buffer containing 1 M sorbitol, 0.1 M EDTA, 0.1%, v/v, β-mercaptoethanol and 100 U ml^-1 ^lyticase (ICN), in a volume 1/5 that of the volume of the of the original cell culture. The digestion proceeded for 10–20 minutes at 30°C. Adequacy of the digestion was monitored by testing a small drop of cell suspension in SDS for viscosity [13]. Mixtures were centrifuged at 800 × g and RNA was isolated by using the QIAGEN total RNA kit, following the manufacturer's protocol. RNA was precipitated with isopropanol, and either used immediately or stored in ethanol at -20°C. Polyadenylated RNA selection was carried out by following the Oligotex mRNA kit protocol (Qiagen).

### Northern blot analysis

Samples of 50 ng of poly(A) RNA were electrophoresed on 1.2% formaldehyde agarose gel blotted to nylon membranes. A 262 bp long fragment of 18S was generated from reverse transcribed total RNA with the primers 5'-TCGATGGAAGTTTGAGGCAA-3' (P1) and 5'-ATTCAATCGGTAGTAGCGACGGGC-3' (P2) based on the previously published sequence (Barnes et al., 1991). After cloning into pCR2.1 (Invitrogen), and sequencing (T7 Sequenase 2.0 kit, Amersham) to confirm that it was 18S specific, the insert was released by *Eco*RI digestion and gel purification, and was labeled with P^32 ^by using random priming. As a control a 499 bp *UB14 *probe was also generated with the primers 5'-GAAGTCGAATCTTCTGACACCATCG-3' (P3) and 5'-TGGTGGAATACCTTCTTTGTCTTGG-3' (P4). The primer design was based on the *UB14 *sequence (Accession No. Z54197) reported by the Stanford Candida Genome project's World Wide Web site [14]. This amplified product was also cloned and sequenced to confirm its specificity. To assess the quality of our RNA, a 5S specific probe was generated with the primers 5'-GGTTGCGGCCATATCTAGCAGAA-3'(P5) and 5'-AGATTGCAGCACAATAGTTTCGC-3' (P6). These primers were based on the reported sequence of the 5S component of *Candida albicans *rRNA [15]. Phosphorimaging volume report analysis (Molecular Dynamics, Sunnyvale, CA) has been employed to quantify objectively the intensity of individual bands. We estimated the percentage of polyadenylated 18S, from phosphorimaging data derived from Northern blots comparing total RNA and poly-A selected RNA (derived from the same amount of total RNA).

### Real-time PCR analysis

To confirm that the polyadenylated form of 18S is increased by five minutes, total RNA was predigested with RNase-free DNase I (New England Biolabs), then reverse transcribed with Superscript II RT (Invitrogen) utilizing an anchored polyT primer 5'-AATTCGGCGAGCTCCGCGGCCGCGTTTTTTTTTTTT-3' (DT12) to generate cDNAs from polyadenylated RNA molecules. The same reaction also contained the primer P6, specific for 5S rRNA subunit that does not get polyadenylated [15]. Using these cDNA templates, a 63 nt long sequence specific for 18S at positions 1585–1648 [5] was amplified by primers P2 and 5'-TCAGCTTGCGTTGATTACGTCC-3' (P7). In a separate reaction, primers P5 and P6 were used to amplify 5S. To insure that there was no genomic DNA contamination, we carried out PCR reactions utilizing primer pairs P2-P7 and P5-P6 on predigested total RNA that was not reverse transcribed and no products were generated (data not shown). Real time PCR reactions were carried out with iQsybr Green Supermix(Bio-Rad) as source of fluorescence, utilizing an iCycler (Bio-Rad) thermocycler. The cycling settings used were; initial denaturation for 3 min at 95°C followed by 40 cycles each consisting of 30s denaturation at 95°C, 30s primer annealing at 55°C and 30s extension at 72°C. Data were analyzed using iCycler iQ version 3.0a.

### Amplification of poly-A extended 18S rRNA

To identify polyadenylation positions involving 18S, total RNA was heated at 70°C for 5 minutes to minimize secondary structures. Reverse transcription was done with the anchored polyT primer DT12, utilizing Superscript II RT (Invitrogen). PCR products were amplified with primers DT12 and P1 which is situated 398 nucleotides from the reported 3' end of the molecule using HotStarTaq (Qiagen). To show the presence of a mature sized polyadenylated molecule, amplification was also carried out with DT12 and 5'-TATCTGGTTGATCCTGCCAGTAGTC-3' (P8) situated at the 5' end of 18S using FailSafe PCR System (Epicentre). Amplified products were subcloned into pCR2.1 (Invitrogen), a number of clones were picked and sequenced (T7 Sequenase 2.0 kit, Amersham). For the full-length clone, only parts of the 5' and 3' ends were sequenced.

## Authors' contributions

HL and CPW carried out and analyzed experiments. JF conceived of the study, designed experiments, analyzed data and wrote manuscript. All authors have read and agree with final manuscript.
